# Preparation of Phosphorylated Chitosan From the Cuttlebone of Sepia pharaonis and the Effect of Concentration on Oral Clinical Pathogens

**DOI:** 10.7759/cureus.69951

**Published:** 2024-09-22

**Authors:** Mukundh Subramanian, Yagniyasree Manogaran, Pasiyappazham Ramasamy

**Affiliations:** 1 Physiology, Saveetha Dental College & Hospitals, Saveetha Institute of Medical and Technical Sciences, Saveetha University, Chennai, IND; 2 Prosthodontics, Saveetha Dental College & Hospitals, Saveetha Institute of Medical and Technical Sciences, Saveetha University, Chennai, IND

**Keywords:** antimicrobial, cephalopods, cuttlebone, innovative, mollusks, phosphorylated

## Abstract

Background: Chitosan, a biopolymer derived from chitin, has attracted scholarly interest because of its antibacterial, biocompatible, and biodegradable characteristics. We can phosphorylate the cuttlebone of *Sepia pharaonis*, a natural source of chitin, to enhance its antimicrobial properties. Phosphorylated chitosan is promising for treating oral infections, which are the causative agents of a variety of dental disorders.

Objectives: The goal of this study is to look into how to make phosphorylated chitosan from cuttlebone and what effect different concentrations have on killing oral clinical pathogens like *Streptococcus mutans*, *Pseudomonas aeruginosa*, *Escherichia coli*, and *Candida tropicalis*.

Materials and methods: We extracted chitin and chitosan from the cuttlebone of a specimen of *S. pharaonis*. We then synthesized phosphorylated chitosan by phosphorylating chitosan. We then assessed the antimicrobial activities of phosphorylated chitosan using the well diffusion method. We characterized and evaluated it using Fourier transform infrared spectroscopy (FTIR), Fourier emission scanning electron microscopy (FESEM), and X-ray diffraction (XRD).

Results: Phosphorylated chitosan, in 100% concentration, had the highest inhibition zone of 14 ± 0.82 mm against *P. aeruginosa* and *E. coli* (14 ± 0.75). However, the two different concentrations studied showed no activity against both *Candida tropicalis* and *S. mutans*.

Conclusion: This work successfully used the cuttlebone of *S. pharaonis* to yield phosphorylated chitosan, subsequently demonstrating its antimicrobial potential against dental clinical pathogens. Different concentrations of phosphorylated chitosan strongly controlled its antimicrobial activity, with larger concentrations exhibiting stronger inhibitory effects. According to these findings, phosphorylated chitosan appears to be a promising material for dental care solutions that target clinical bacteria in the mouth.

## Introduction

The main component of living things like fungi and crustaceans, chitosan, is the completely or partially deacetylated form of chitin. It comprises 2-amino-2-deoxy-β-d-glucopyranose and 2-acetamido-2-deoxy-β-d-glucopyranose groups. This polymer is generally known to be recyclable and harmless through enzymes. Its commercial, sustainable, and biological applications have received a lot of interest in recent decades. Researchers claim that chitosan and its derivatives offer beneficial biological uses such as anti-microbial, anti-cancer, anti-cholesterol-emic, blood anti-coagulant, wound treatment, and coverings [1]. Given that the chemical alteration of chitosan does not alter the substance's basic structure, preserves its original physical, chemical, and biological properties, and ultimately yields novel characteristics based on the type of group established, there is significant interest in creating new bifunctional materials. Because of the intriguing biological and chemical characteristics of these molecules, a number of methods for obtaining phosphate equivalents of chitosan have been developed. Among all, it has metal-chelating [2] and antimicrobial [3] qualities.

Research has demonstrated that the addition of groups, such as phosphonic acid or phosphonate, to chitosan through the interaction of a phosphorylating agent onto the amino groups enhances its chelating properties and potentially modifies its ability to dissolve [4]. Scholars have conducted research on the phosphorylation of hydroxyl functionalities in chitosan to yield phosphonate using two primary methods. On the one hand, methane sulphonic acid is present during the reaction between chitosan's hydroxyl functions and phosphorous pentoxide's roles [5]. Conversely, urea causes a reaction between phosphoric acid and the hydroxyl functionalities of chitosan [6]. Healthcare, metal binding, pharmaceutical delivery, and other domains mostly use these derivatives [7]. Chitosan can also be interpolymer linked with triple phosphate or polyphosphate to yield phosphate compounds [8].

Numerous researchers have found that the degree of deacetylation (DDA) and the molecular weight (MW) of chitosan determine its antimicrobial properties. MW and DDA increase the solubility of chitosan, as well as their interaction with the cell walls of the target microorganisms. As a result, chitosan derivatives' antimicrobial qualities differ. Few studies have been conducted on the antimicrobial activities of chitosan derived from cephalopods, such as cuttlebones, despite the extensive exploration of chitosan derived from fungal cell walls and crustacean shells [9]. The main obstacle to chitosan's use in biological systems is its insolubleness in water. It has proven feasible to overcome the insolubility by producing phosphorylated chitosan that is soluble in water. Recovering the waste that is generated during the processing of seafood can be highly profitable due to the abundance of high-value materials such as chitin. After converting chitin into chitosan and phosphorylating it with orthophosphoric acid, In vitro techniques also assessed the antimicrobial activity [9,10]. Therefore, we planned the present investigation to synthesize phosphorylated chitosan from the cuttlebone of *Sepia pharaonis*; characterize its structure through Fourier transform infrared spectroscopy (FTIR), field emission scanning electron microscopy (FESEM), and X-ray powder diffraction (XRD); and study its *in vitro* antimicrobial potential against oral clinical pathogens.

## Materials and methods

Collection and preparation of samples

Cuttlebone of *S. pharaonis* was collected from the Cuddalore Fish Landing Center, Tamil Nadu, India (Lat. 11°42’ N; Long. 79°46’E), during the period of January 2023. The cuttlebone was washed, dried, and pulverized to extract the chitin and chitosan.

Extraction of chitin and preparation of chitosan from the cuttlebone of *S. pharaonis*

We removed minerals, separated proteins, and decolored the cuttlebones to remove chitin. We mixed a thousand milliliters of 10% by weight HCl (1.04 M) with 100 g of powdered cuttlebone and left the mixture for 24 h at 25 °C to achieve demineralization. After filtering with filter paper, we washed the residue with neutral distilled water. We subsequently submerged the residue in 1,000 milliliters of 10% by-weight sodium hydroxide (2.5 M) for 24 h at 60 °C to deproteinize it. We used filtration to remove the proteins and neutrally washed the residue with distilled water. The mixture turned clear and colorless. Subsequently, the cuttlebones underwent the previously mentioned process twice. We extracted and dried ethanol-soluble materials using 250 milliliters of 95% ethanol to obtain crude chitin. We dried 200 g of chitin in an air oven at 50 °C for the entire night [11]. NaOH aqueous solution (40%) at 110 °C underwent a deacetylation procedure to transform chitin into chitosan [12].

Synthesis of phosphorylated chitosan

We made phosphorylated chitosan by dissolving 2 grams of crushed chitosan in 30 grams of urea and 50 milliliters of DMF. We then added 5.2 milliliters of orthophosphoric acid to the chitosan mixture. We treated the mixture at 150 °C for one hour. We cooled the reaction mixture, re-immersed the residue in distilled water, precipitated the entire mixture, and completely cleaned it with methanol. The pH was changed to 10-11. We employed a 12,000 Da MW cut-off dialysis membrane to dialyze the solution against distilled water for a duration of 48 hours. We then lyophilized the product to produce phosphorylated chitosan [13].

Characteristic analysis of phosphorylated chitosan

FTIR for Phosphorylated Chitosan

A Brukers Alpha II FTIR (Berlin, Germany) was used to evaluate the phosphorylated chitosan that we isolated from *S. pharaonis *using a spectrum analyzer.

FESEM for Phosphorylated Chitosan

We used the INCA EDS scanning electron microscope (Hitachi High-Technologies, JEOL-JSM-5610LV, Tokyo, Japan) to examine the surface topography and microstructure of the chitosan. We coated the sample with a thin layer of gold/palladium (40/60) through direct alloy condensation at 20 V, using the Hitachi Hus-4 vacuum evaporator. We assessed the preparation using an accelerating potential of 0.5-30 kV at different magnifications.

XRD for Phosphorylated Chitosan

The angle of diffraction 2 and the specimen orientation determined the amplitude of the reflected X-rays (XRD-6000 Shimadzu). This diffraction pattern allowed for the determination of the specimen's crystalline phases, extremely precise measurements of its structural features, and the dimensions and orientation of crystallite-small crystalline areas.

In Vitro Antimicrobial Activity

We used three species of bacteria and one species of fungi as test organisms (bacterial strains: *Streptococcus mutants*, *Pseudomonas aeruginosa*, and *Escherichia coli*; fungal strains: *Candida tropicalis*). We prepared the media for Sabouraud dextrose broth and nutrient broth and then autoclaved them for 15 minutes at 15 lbs of pressure. After sterilizing the nutrient and Sabouraud dextrose broth, we separately introduced each strain and cultured it for 24 hours at 37 °C. We prepared Hinton agar, Sabouraud dextrose agar (SDA), and brain heart infusion agar (BHI). We prepared the agar using a sterile cotton swab, autoclaved it for 15 minutes at 15 lbs of pressure, and then poured it into sterile Petri dishes. We inoculated the agar Petri dishes with stationary-phase cultures using a sterile cotton swab. Using the agar-well diffusion method, the antimicrobial activity was determined against four pathogenic isolates [14]. Bacterial and fungal nutrient broth and Sabouraud dextrose broth cultures were grown on clean MHA, SDA, and BHI agar plates for 24 hours. We created aseptic wells with a 5-mm diameter in the inoculation plates. We generated four distinct amounts of phosphorylated chitosan (1.25-5 mg/mL) by dissolving it in the appropriate solvent and then added it to the wells. We filled the marked wells with standard (fluconazole, streptomycin, ciprofloxacin, and chloramphenicol-1 mg/ml) and control (0.2% distilled water). We kept the plates upright and incubated them at 37 °C for 24 hours. We measured the zone of inhibition and conducted the experiment in duplicate.

## Results

FTIR spectral analysis

**Figure 1:**
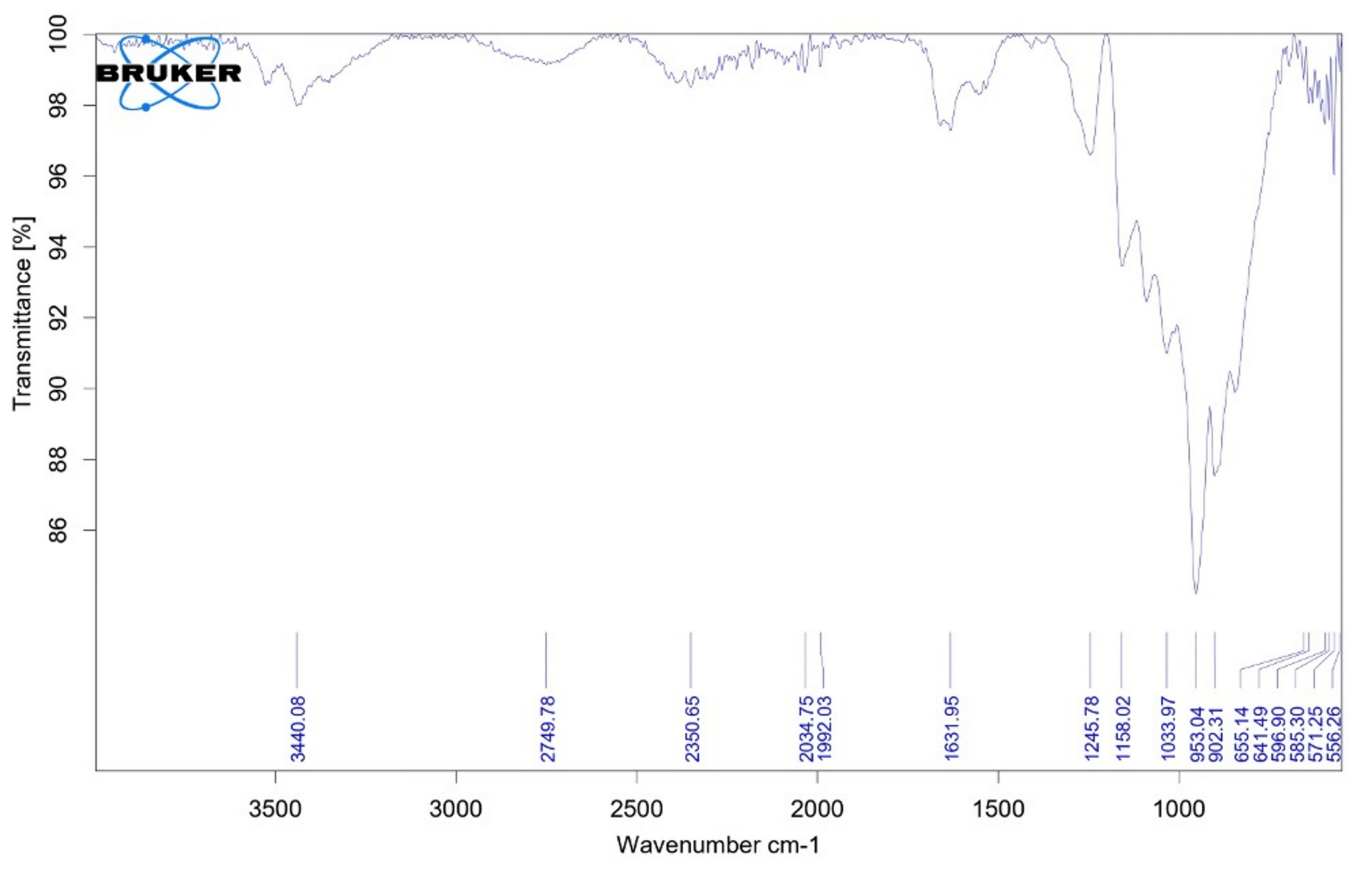
FTIR analysis of phosphorylated chitosan from the cuttlebone of Sepia pharaonis

The IR spectra at 3440.08 cm^-1^ show broad, strong bands that indicate the presence of hydroxyl groups (OH) in the sample. In this case, phosphorylated chitosan connects to these hydroxyl groups. Chitosan, a natural biopolymer made from chitin, acquires new functional groups, such as phosphate esters, when it is phosphorylated or provided with an additional phosphate group. A larger absorption peak is the result of a strong hydrogen bond between the hydroxyl groups, as evidenced by the bands' broadening. We observed weak bands of absorption at 2350.65 cm^-1^ (Figure 1). The FTIR spectrum reveals the CH_^2^_ stretching vibrations of the CH_2_ groups through the appearance of moderate absorption bands at approximately 2350.65 cm^-1^. This implies the sample contains methylene groups (CH_2_). Since methylene groups are frequently found in organic compounds, their presence here suggests the existence of a hydrocarbon chain, which is most likely a related or essential component of the chitosan structure. The FTIR spectrum data indicates that the sample contains phosphorylated chitosan with hydroxyl and CH_2_ groups. In the domains of chemistry and materials science, FTIR is a crucial instrument for identifying functional groups in a variety of chemicals, polymers, and biomolecules. It helps scientists identify and understand the chemical makeup and structure of materials.

FESEM analysis

**Figure 2:**
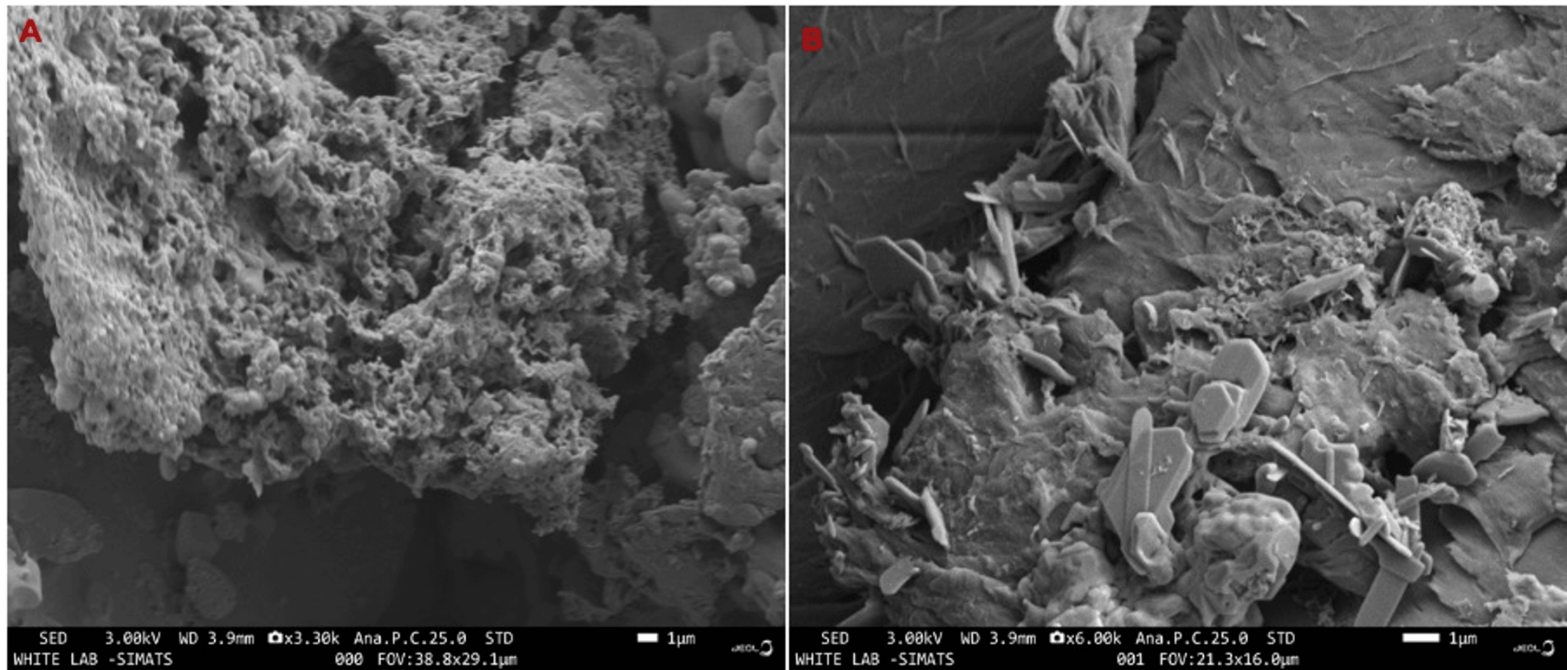
2A, 2B. FESEM analysis of phosphorylated chitosan from the cuttlebone of Sepia pharaonis

Phosphorylated chitosan showed up as porous, chain-like fibril structures in electron micrographs. The SEM image (Figure 2A, 2B) further supported the idea that phosphorylated chitosan was involved in biological uses.

XRD analysis

**Figure 3:**
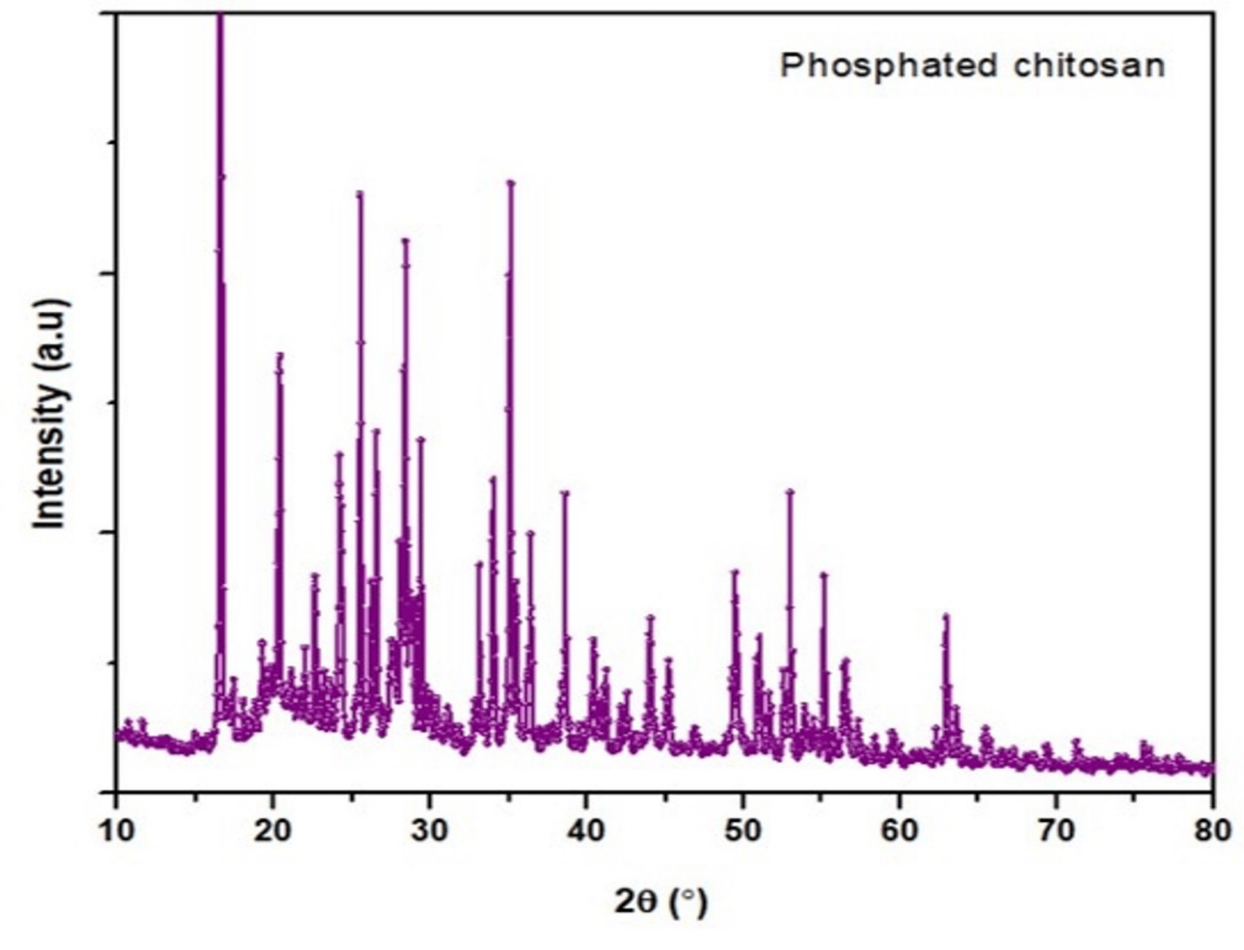
XRD analysis of phosphorylated chitosan from the cuttlebone of Sepia pharaonis

﻿﻿The phosphorylated chitosan's XRD analysis revealed incredibly broad peaks at 2θ=17° (Figure 3). In phosphorylated chitosan, weak peaks were seen at 2θ values of 19°, 26°, 31°, 36°, 53°, and 64°. However, in phosphorylated chitosan, the rather broad peak at 2θ=17° weakened and the peak at 2θ=64° disappeared. These findings imply that chitosan is quite compatible. The phosphorylated chitosan exhibits an amorphous form, according to the XRD pattern.

In vitro antimicrobial activity

**Figure 4:**
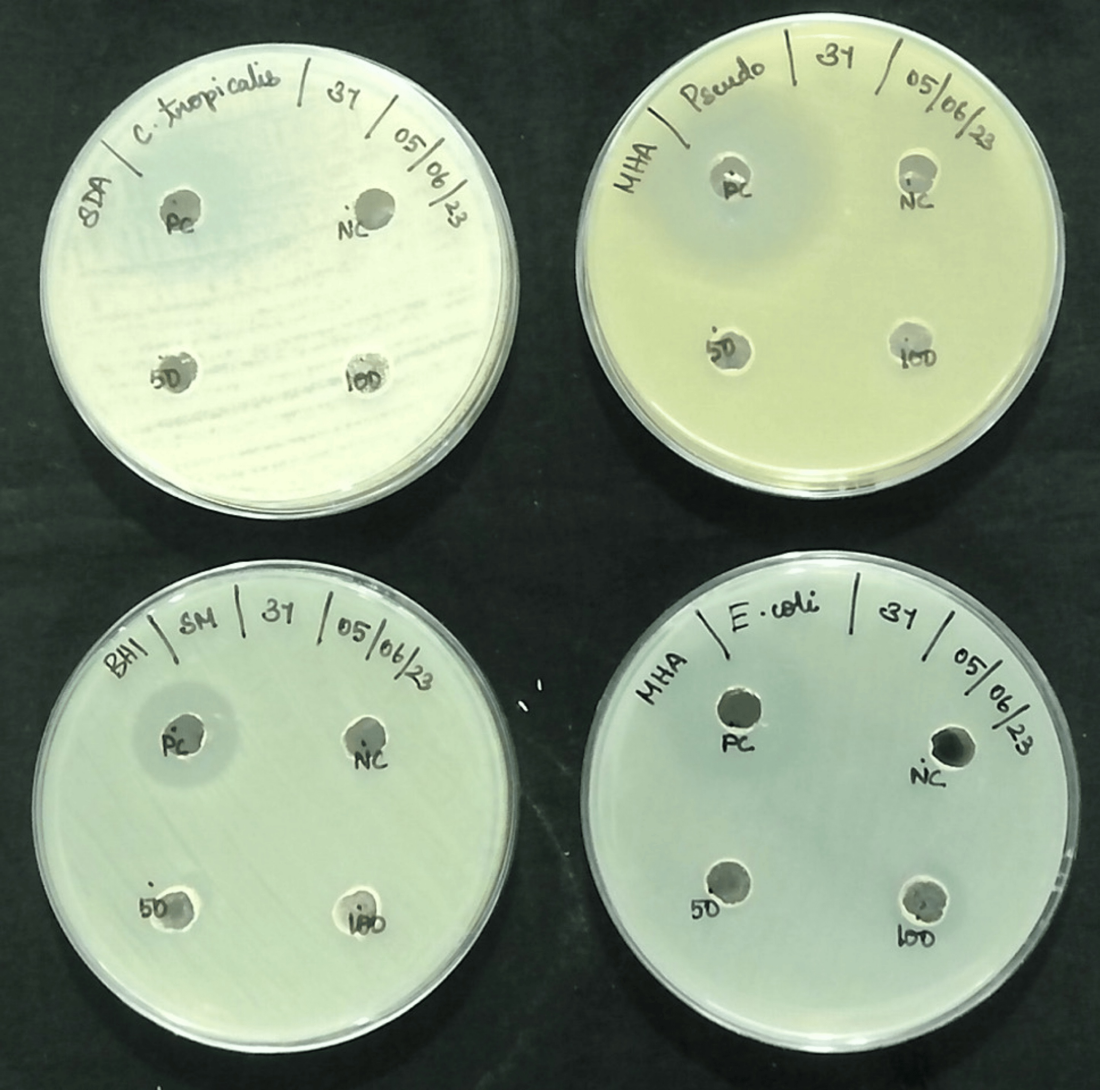
Antimicrobial activity of phosphorylated chitosan from cuttlebone of Sepia pharaonis by the well diffusion method

The antimicrobial activity of phosphorylated chitosan from *S. pharaonis *against bacteria and fungi using the well diffusion method. Figure 4 illustrates how water-soluble phosphorylated chitosan can create a zone of inhibition to stop the growth of test bacteria and fungi on solid media (Table 1). High-concentration phosphorylated chitosan exhibited the greatest inhibitory zone of 14±0.82 mm against *P. aeruginosa* and 14±0.75 mm against *E. coli*. Nevertheless, the two distinct doses investigated showed little efficacy against both *C. tropicalis* and *S. mutans.*

**Table 1:** Antimicrobial activities of phosphorylated chitosan from Sepia pharaonis

S. no.	Bacterial/fungal strains	Phosphorylated chitosan from the cuttlebone of *Sepia pharaonis*		+C (mm)	-C (mm)
		50% (mm)	100% (mm)		
1	Candida tropicolis	-	-	34±0.82	-
2	Streptococcus mutans	-	-	23±1.53	-
3	Pseudomonas aeruginosa	-	14±0.82	30±1.75	-
4	Escherichia coli	-	14±0.75	25±2.25	-

## Discussion

Chitin undergoes partial deacetylation to produce chitosan, a cationic polymer that is both biocompatible and non-toxic. The main focus is on chemically changing chitin and chitosan to make new functionalized materials [15]. This method keeps the materials' original physical, chemical, and biological properties while adding new groups without changing their basic structure. Pharmaceuticals can chemically alter the amino and hydroxyl groups of chitin and chitosan at specific locations to create compounds that dissolve at neutral pH. Numerous studies have reported a yield percentage of phosphorylated chitosan, despite the scarcity of such studies. Researchers produced 76% of the phosphorylated chitin. The study yielded *S. lessoniana* gladius chitin [16]. Researchers reported that *S. lessoniana*, *S. inermis*, and *S. aculeata* contributed 90%, 80%, and 90.3% of the phosphorylated chitosan [17].

However, the present study found that 81% of the phosphorylated chitosan cuttlebone of *S. pharanonis* was reported. The degree of deacetylation and substitution of phosphorylated chitosan was a major factor in its solubility in water. Typically, phosphorylated chitosan with a low to medium degree of substitution and deacetylation would dissolve readily in water. When polyampholytes try to dissolve phosphorylated chitosan with high levels of deacetylation and substitution, they cannot. This might be because phosphate and amino groups form a polyion complex, which is a salt linkage between molecules [18]. The difference between the solubility rate and yield percentage may be due to the amount of chitosan deacetylation and phosphate group substitution.

The FTIR of phosphorylated chitosan to confirm the structural change that had occurred. The FTIR spectrum of phosphorylated chitosan from gladius of *S. lessoniana* was recorded between 462.31 and 3409.55 cm^−1^. In this investigation, the peaks showed were lying between 641.49 and 3440.08 cm^−1^. The peaks at 3440.08, 2749.78, 2350.65, and 2034.75 cm^−1^ show the structure of phosphorylated chitosan. These points correspond to OH and H-bonded NH2stretching, aliphatic CH stretching, amide C=O stretching, and NH bending, in that order. The phosphorylated chitosan spectrum shows that the peaks at 1033.97 and 556.26 cm^−1 ^are caused by the P-OH group. The P=O stretching peak at 1245.78 cm^−1^ shows that phosphate groups were present in the original chitosan. This conclusion was aligned with the findings of other researchers [9], who also claimed that P=O stretching was responsible for the large peak observed at 3500 cm^−1^. The P-OH group was the cause of the peaks observed at 500 cm^−1 ^and 1050 cm^−1^.

Numerous products, such as films, microspheres, and nanoparticles, can be made using chitosan. The SEM is to examine the distribution, size, and shape of the particles within these structures. This information improves the manufacturing processes and customizes the chitosan components for their intended uses. SEM imaging is used to compare different chitosan formulations, processing techniques, or modifications. By visually analyzing differences in surface shape and framework, scientists can improve on current formulations or identify which chitosan substances are most appropriate for a given application. The phosphorylated chitosan’s SEM image displays an intriguing microstructure made up of a web of linked fibers or particles. It appears to have an even outer layer covered in holes and flaws [19].

Both phosphorylated and untreated chitosan membranes revealed a uniformly smooth film surface. Unlike previous studies that used the H_3_PO_4_/urea/DMF technique for the same objective, the current investigation did not discover blisters or air bubbles following chemical treatment. The chitosan membranes' cross-section revealed layers of fibrils clearly aligned with the surface. We demonstrated that completely deacetylated chitosan forms ultrastructures with a rough cross-section devoid of fibrils. Conversely, the total N-acetylation of the first form results in a random arrangement of fibrils. To find out why the filaments were there, linked them to partly N-acetylated chitosan chains that were squeezed together to make the membranes during the opening-up phase. Phosphorylated chitosan frequently displays diffraction peaks that align with its amorphous regions. The peaks' positions and strengths revealed details about the molecular organization and order inside the chitosan structure.

Chitosan with 85% deacetylation and its oligosaccharides killed pathogenic microorganisms more effectively than non-pathogens [20]. Researchers tested newly created water-soluble chitosan derivatives for their antimicrobial properties against various types of bacteria, including *S. aureus*, *B. subtilis*, *P. aeruginosa*, and *S. mutans* [21]. Chitosan and its water-soluble derivatives exhibited concentration-dependent antimicrobial efficacy against all pathogenic strains. Furthermore, the findings demonstrated a relationship between the compounds' antimicrobial activity and concentration, with higher concentrations corresponding to increased antimicrobial activity. The results of this investigation are in line with those of [22], who showed that chitosan's antibacterial and antifungal properties increased with concentration.

It is important to take into account the chemical and structural characteristics of chitosan when evaluating its inhibitory efficacy against bacteria and fungi. Since the external layer of bacteria and fungi serves as an effective outer permeability barrier against macromolecules, chitosan, being a polymer, is unable to pass through it. Thus, it is unlikely that chitosan will be able to directly access the internal components of the cells. Chitosan has a pKa value of less than 6.3 and a positive charge at the C2 position of the amino group. This makes it a multi-cationic structure that interacts with the lipopolysaccharides and surface proteins of bacteria [23]. Moreover, the cationic group binding and adsorption do not work as well because the chitosan derivative is mostly negatively charged when the conditions are neutral. Given a pH value, the degree of NH_2_ protonation in chitosan remains constant [24]. As pH levels rise, the degree of NH_2_ protonation decreases. We conducted the anti-microbial test using sterile distilled water, which allows the amino group to be unrestricted and exhibit excellent coordination abilities.

Researchers have demonstrated chitosan's antimicrobial activity against a diversity of bacterial, filamentous fungal, and yeast strains. Still, chitosan's ability to kill microbes depends on the type of microbe, the pH of the solution it is in, and the molecular mass and structural percentage of the chitin units in the polymeric chain [25]. Chitosan derivatives' antimicrobial action is closely linked to the development of hydrophobic micro-urea. Strong intramolecular and intermolecular hydrogen bonds create hydrophobic micro-urea in the polymer chain [26]. This is because NH_2_protonation is very low at pH 7, which makes NH_3_^+^ repel it weakly. The polymer chain's carboxyl group is also very hydrophilic at the same time. Consequently, there are hydrophobic and hydrophilic segments in the polymer chains. The amphiphilic structure makes it easier for the chitosan derivative to attach to the cell walls of bacteria.

*S. kobiensis *produced phosphorylated chitosan, which at various doses demonstrated antimicrobial activity against *P. aeruginosa* and *E. coli*. However, it did not show any activity against any of the tested isolates of bacteria [27]. This study, on the other hand, found that 100% phosphorylated chitosan had stronger antibacterial activity. *C. tropicalis* and *S. mutans* showed no zone of inhibition, while *P. aeruginosa* and *E. coli *showed a clear 14-mm zone of inhibition. This shows that phosphorylated chitosan from *S. pharaonis* was better at killing bacteria than phosphorylated chitosan from *S. kobiensis*, even at different doses. However, their efficacy in eliminating fungus was somewhat limited.

The precise mechanism behind the antimicrobial effect of chitosan derivatives remains unclear, despite various hypotheses. Water-soluble chitosan made cell membranes more permeable, which eventually caused bacterial cell membranes to rupture and discharge their contents [28]. The microbial cell surface may precipitate and stack the water-insoluble chitosan molecules, creating an impermeable coating that encloses the cell and obstructs the channels that are essential to live cells. We think that this layer will stop essential solutes from getting through and could permanently weaken the cell wall, which will let a lot of cell parts leak out and eventually kill the cell [29].

The antimicrobial effect of phosphorylated chitosan may have originated from making cell membranes more permeable, which eventually broke bacterial and fungal cell membranes and let their contents out. Finding out how structurally modified, phosphorylated chitosan exhibits anti-microbial activity was the goal of this investigation. The findings further support the theory that phosphorylated chitosan's phosphate group position improves the antimicrobial activity's efficacy. A clear barrier zone formed around phosphorylated chitosan made from *S. pharaonis* cuttlebone. This barrier was effective against both fungal and bacterial types. Data from the literature indicated that phosphorylated chitosan has superior antimicrobial properties than chitosan. Indeed, our findings were overwhelmingly positive, demonstrating a robust antimicrobial capacity even when we took into account the different sequences under investigation. Hence, phosphorylated chitosan made from cuttlebone chitosan, specifically from *S. pharaonis*, can be used instead of chitosan because it kills microbes.

Limitations

This study only used one species, *S. pharaonis*, so it is likely that other chitosan sources cannot apply the findings. Variations in the extraction and phosphorylation processes can also affect yield and efficiency, making the results challenging to replicate. We need additional in vivo research to verify the effectiveness of antimicrobial assays, as these in vitro methods may not perfectly replicate the complex physiological parameters found *in vivo*. This study did not discuss important factors like immunogenicity and toxicity that are crucial for the therapeutic application of phosphorylated chitosan. The last item overlooked was the viability and economics of manufacturing phosphorylated chitosan from *S. pharaonis* cuttlebone.

## Conclusions

This work successfully used the cuttlebone of *S. pharaonis* to create phosphorylated chitosan, which then demonstrated anti-microbial activity against dental clinical infections. We found that the concentration of phosphorylated chitosan strongly controlled its antibacterial and antifungal activity, with larger concentrations exhibiting stronger inhibitory effects. According to these findings, phosphorylated chitosan appears to be a promising material for dental care solutions that target clinical bacteria and fungus in the mouth.
